# Gamification and Soft Skills Assessment in the Development of a Serious Game: Design and Feasibility Pilot Study

**DOI:** 10.2196/45436

**Published:** 2023-07-26

**Authors:** Luca Altomari, Natalia Altomari, Gianpaolo Iazzolino

**Affiliations:** 1 Department of Mechanical, Energy and Management Engineering University of Calabria Rende Italy; 2 Department of Mathematics and Computer Science University of Calabria Rende Italy

**Keywords:** gamification, soft skills, recruitment, serious games, assessment, process mining, work-life skills

## Abstract

**Background:**

The advent of new technologies has had a profound impact on the labor market, transforming the way we work and interact with each other. With the rise of digital tools and platforms, gamification has emerged as a powerful technique for enhancing productivity and engagement in various fields, including human resource management. In particular, gamification has been found to be effective in developing and assessing soft skills, which play a critical role in determining the success of individuals, teams, and organizations.

**Objective:**

We present a serious game that identifies the most sought-after skills in the job market and offers feedback, and we provide a set of guidelines for the creation of serious games.

**Methods:**

We present the serious game Among the Office Criticality (AOC). The AOC game structure involves a set of sequence analysis techniques, which is known as process mining.

**Results:**

The pilot study findings indicate that the game is both engaging and beneficial to subjects, suggesting that the results align with current theoretical perspectives. Furthermore, the study suggests that the obtained data can be extended to the broader population.

**Conclusions:**

This study illustrates a serious game structured according to the needs of the labor market and developed to put the user at the center, using evaluation techniques consistent with the literature, with the aim of constituting an interdisciplinary approach suitable for adequately assessing users and creating value for them.

## Introduction

### Background

The field of human resources has transformed traditional and outdated personnel selection methods through technological advancements, paving the way for more technologically advanced methods that reduce hiring costs and optimize the evaluation of candidates during the recruitment process. This is particularly relevant when it comes to millennials and Generation Z. Gamification is among these methods. It can be defined as “a package of services in which a basic service is enhanced by rule-based service systems that provide the user with feedback and interaction mechanisms that aim to facilitate and support users’ global value creation” [1], and in recent years, it has become an increasingly popular trend in nongaming contexts [2] to increase motivation and engagement [3]. Gamification involves the use of structured rules and competitive conflicts to achieve specific goals, and in which a person is fully engaged with the context, losing track of time and experiencing the thrill of doing rather than achieving external rewards [4]. Research has shown that play and games can be linked to positive social behavior [5], the development of important skills such as self-regulation and empathy [6], and the promotion of teamwork [7].

Gamification is often used to enhance services and test gaming experiences and can be a powerful tool for influencing behavior [1]. However, some studies have examined issues related to gamification [8]. This technique is applied in settings related to learning, business, or promoting a product or service [9]. Through elements, such as competition, goal achievement, and reward, gamification can increase users’ motivation, engagement, and satisfaction in an activity [10,11].

Many researchers have suggested that gamification and game-based assessments could predict work performance as an alternative to traditional selection methods [12,13]. Fetzer et al [13], according to the concept of “stealth assessment,” highlighted the potential of game-based assessments for predicting work performance. Invisible evaluation can accurately and efficiently detect a subject’s level of competence, can continuously extract data on their performance during the game [14], and is almost undetectable, reducing test anxiety while maintaining validity and reliability [15]. In this context, a gamified assessment environment could distract candidates from being assessed, promoting unconscious rather than desirable or socially acceptable behavior. Assessment and learning tools that may be created through the use of gamification are known as serious games [16], which are games designed for a primary purpose other than pure entertainment [17], like supporting human capital development [18]. Serious games are designed for educational, training, or informational purposes that combine the playful experience with learning specific skills and competencies [19]. Unlike traditional games, serious games were created to impart knowledge and teach players how to apply those skills in real life by creating engaging and entertaining experiences that prompt users to perform certain actions, improving the effectiveness and efficiency of those activities.

Various sectors, such as education [20], health care [21], mobility [22], marketing [23], and the environment [24], have been equipped with this innovation, simulating situations in which the player must make decisions, solve problems, or develop specific skills. Moreover, owing to the interactive nature of serious games, players can learn actively, receiving immediate feedback on their progress and the correctness of their actions [25].

If the game is user-friendly and realistic, it tends to generate positive responses from candidates [26]. Thus, one of the cornerstones of these games (as well as an aspect this paper will focus on) is the development of awareness related to one’s skills, particularly those skills called “soft skills,” which are indispensable in the world of work [27].

In relation to the connection between gamification and transversal skills, game elements, such as textures, feedback, avatars, visual effects, and voiceovers, can be effectively used to assess candidates’ soft skills [28].

Problem-solving skills, strategic thinking, and adaptation to new contexts are soft skills, also known as “behavioral skills.” They are non-technical skills (as opposed to hard skills) important for personal and professional success. Using game mechanics, gamification can make learning these skills more engaging and effective. Serious games play a key role in developing soft skills (such as problem solving, planning, and analytical thinking) in the world of work [27]. Certain elements, such as a risk-free environment, predispose users to a setting conducive to experimentation and exploration, activating stimuli, such as curiosity, learning by discovery, and perseverance [29].

Serious games are playful and interactive tools often used in education with pedagogical goals, which are designed to teach skills, notions, and strategies [30,31]. Serious games offer active involvement, immediate feedback, experiential learning, collaborative learning, and learning personalization, which enhance positive learning effects [32]. The scenarios designed for serious games have a positive relationship with student engagement and academic performance [33]. Despite their potential, serious games still pose a challenge to the educational world, and there is a need for more emphasis on less formal and traditional models that are suitable for teaching today’s generation of students [34]. Serious games are an important resource for the world of training and education, facilitating the learning of specific disciplines.

Transversal skills often overlap with digital skills, which have become increasingly necessary for performing tasks due to the growth of technology in recent years. Digital skills involve the use of information and communications technology (ICT) tools and can be classified as operational, formal, informational, or strategic skills [35].

Based on these premises, this research proposes an innovative method for addressing an issue dear to companies, the recruitment process. Recruiting an unsuitable candidate can be very costly, both in terms of money and nonproductive time. Therefore, many companies are looking for innovative ways to improve their recruitment process to attract and identify the most suitable candidates for available positions. This involves the use of cutting-edge recruiting techniques and an increased focus on assessing candidates’ skills and qualities [36].

Psychological tests that measure cognitive abilities and personality tests, which are traditional selection methods, can somewhat predict job performance [37]. As previously mentioned, the game is an attractive and valid alternative to traditional selection methods, and gamifying these tests can be a way to assess candidates with the benefits stated above.

The COVID-19 pandemic has prompted a shift in the labor market toward Industry 5.0 to the New Normal Era, a time of collaboration between advanced technology and human creativity to address business, social, and environmental challenges [38]. This has had an impact on the labor market, affecting both demand and requirements, as well as leading to the emergence of the “great resignation” phenomenon, in which individuals across countries and industries are exhibiting a heightened awareness of work conditions and opportunities [39,40].

To successfully adopt artificial intelligence (AI), organizations and individuals must embrace transformative changes in their practices, recognizing the foundational role of connected technologies in driving increased digitization in business and management worldwide. Key factors for successful AI adoption include team coordination, organizational culture, governance strategy, and AI-employee integration strategies. Furthermore, the integration of serious games and AI provides new computational tools for social research. Soft skills, such as communication, problem solving, critical thinking, and creativity, are crucial for successful AI adoption, while leadership plays a vital role in setting a vision and strategy for its implementation [41,42].

Recent studies have emphasized the importance of soft skills and talent in the New Normal Era, where work has changed significantly and new methodologies, such as smart working and remote working, are becoming increasingly prevalent. As a result, soft skills are now a top organizational priority [43].

### State of the Art

#### Industry 5.0: Impact of Emerging Technologies on Gamification and the Labor Market

With the increasing adoption of new technologies, such as AI, virtual reality, metaverse, blockchain, augmented reality, and collaborative robots, it is crucial to understand the transition from Industry 4.0 to Industry 5.0 for creating sustainable business and management practices. Industry 4.0 was characterized by automation technologies, the Internet of Things, and smart factories, while Industry 5.0 involves the collaboration between advanced machinery and human creativity to solve business, social, and environmental issues. The adoption of transformative changes in practices is essential for organizations and individuals to understand as these connected technologies provide the foundation for increased digitization in business and management worldwide. While the use of serious games and gamification has become increasingly popular in recent years, some of these emerging technologies, particularly AI, have the potential to be disruptive for the world of work and have a significant impact on human resources, recruitment, and corporate life [38].

Team coordination and organizational culture are also crucial elements that organizations must consider when adopting AI. Effective coordination between team members is essential for successful AI implementation, and organizational culture plays a critical role in supporting this coordination. An innovation mindset is also necessary to foster a culture of experimentation, learning, and continuous improvement, which are all essential for successful AI adoption.

Governance strategy is another key area that organizations must consider when adopting AI. Effective governance ensures that AI is being used ethically, responsibly, and in compliance with legal and regulatory frameworks. AI-employee integration strategies are also important, as they help to ensure that employees feel comfortable working with AI systems and are equipped with the necessary skills to do so [41].

Pérez et al [42] suggested that the integration of serious games and AI offers new computational tools for social research. By developing and applying gamification with an eye toward both design and efficacy, organizations can increase productivity and promote better decision-making processes in the workplace. The use of gamification and serious games in the workplace can be a promising tool to enhance motivation, engagement, and decision-making processes among employees.

An evidence-based approach to gamification considers its effectiveness to be dependent on the use of scientifically proven principles and tactics that can impact outcomes [44]. Optimistic results have been found in the integration of some of the newest technologies, as AI, virtual reality, and serious games are being increasingly used in several industries, such as the healthcare industry where there was a great effort concerning new technologies and research during the COVID-19 pandemic, yielding favorable outcomes for the treatment of various health conditions as well as for medical education and training [45-47]. Moreover, Schönbohm and Zhang [48] revealed the effectiveness of serious games in facilitating strategic decision-making in the context of the COVID-19 pandemic.

In the era of the new normal, the global workforce is experiencing an unprecedented phenomenon known as the “great resignation.” This phenomenon is characterized by individuals in various countries and industries exhibiting a heightened awareness of work conditions and opportunities, which is indicative of an overall increase in people’s awareness of work. However, this phenomenon has also brought about uncertainty, challenges, and opportunities for organizations due to external factors such as the gig economy and the implementation of technology [39,40].

Recent research by Duch-Brown et al [49] investigated the relationship between market power, AI, and online labor markets. They showed that market-designing platform providers can influence labor demand and supply elasticities on online labor markets by setting specific terms and conditions on the platform. The study also revealed a significantly higher demand for AI-related labor and a significantly lower supply of AI-related labor, indicating the need for further exploration of the role of AI in online labor markets. Similarly, Deranty and Corbin [50] highlighted the potential societal impacts of AI deployment in work, particularly in the areas of technological unemployment and wealth polarization.

This presents new challenges and opportunities for soft skills [51], as well as technical skills and fusion skills [52], which are those skills and attributes required for individuals to thrive in the fusion environment of the 21st century. Therefore, staying updated with technological advancements and market-specific demands is essential for individuals employed in data science–related fields (more so than others) to enhance and maintain their employability, especially in the digital renaissance. Additionally, real-time identification of market demands plays a crucial role in balancing the adequate supply and demand of talent in this era [53].

#### The Future of Work Relies on Soft Skills in the New Normal Era

A new technological revolution is going on, and many organizations are eager to adopt new technologies in order to gain a competitive advantage. However, implementing new tools, like AI-based tools and technologies, is not enough to achieve success. A recent review on human resource management conducted in March 2023 revealed that organizations need to look beyond technical resources and focus on developing nontechnical resources as well [41]. The review highlights several nontechnical resources that are critical to successful AI adoption. These include human skills and competencies, such as communication, problem solving, critical thinking, and creativity, which are essential for effective collaboration with AI systems. Additionally, leadership is vital for setting a vision and strategy for AI adoption and ensuring that it aligns with the organization’s overall goals and values. In conclusion, the review underscores the importance of developing nontechnical resources in addition to technical resources when adopting AI. By investing in human skills and competencies, leadership, team coordination, organizational culture, innovation mindset, governance strategies, and AI-employee integration strategies, organizations can fully realize the benefits of AI adoption and gain a competitive advantage in the marketplace [41].

The 2023 Pulse of the Profession report by Project Management Institute highlights that power skills, which are soft skills built upon a solid foundation of technical skills, are a top organizational priority [43]. Developing these skills can lead to delivering results that add value to both the organization and its customers.

Redefining work requires more than skill development, as skills have inherent limitations. Simply retraining workers to perform different routine tasks or adapting to new technologies to complete the same tasks does not address the underlying challenges for workers or capture the potential benefits for companies. The vision of redefined work involves shifting workers’ focus from routine tasks to identifying and resolving hidden issues and opportunities. While automation can facilitate this shift, it is not just about automating tasks or using technology. It does not involve changing the workforce composition or retraining employees for different domains, and it does not require employee suggestion boxes, 20% work time for innovation or entrepreneurship, or innovation centers. Workers must develop and use their human abilities to identify issues, solve problems, implement solutions, and iterate to recognize and resolve hidden issues and opportunities. A key characteristic of this work vision is its continuous evolution. Problem identification and solution methods are typically used to correct processes, address deviations, or eliminate inefficiencies, with the goal of incorporating feedback into more structured and defined work. Loosening the structure is only a temporary measure to advance the process [51].

The job market has undergone a significant transformation due to the pandemic, which has been expedited by the emergence of new technologies. To thrive in this new era, possessing soft skills, such as communication, problem solving, creativity, and collaboration, as well as the ability to adapt to change, is crucial [54], even if digital and technological skills are vital for enhancing a firm’s performance. Moreover, Akrim [55] stressed that while knowledge is important, soft skills cannot be overstated when it comes to securing one’s future.

To gain a competitive edge in the job market, integrating talent and knowledge management strategies is imperative [56], and to succeed in the future of work, prioritizing soft skills and talent management strategies in recruitment and training processes is essential. Identifying unique talents and skills is crucial to gaining a competitive advantage. High demand for soft skills, such as creativity, emotional intelligence, critical thinking, and problem solving, necessitates their recognition and prioritization in organizations’ development efforts [57].

#### The Pedagogical Side of Serious Games

Serious games serve as an effective and result-oriented medium, utilizing interactive and playful forms of engagement [30]. They represent tools designed with the goal of teaching something, such as skills, notions, and strategies, and offer the possibility for the user to identify with learning and formative situations, and thus, it can be said that they pose pedagogical goals [31]. Serious games are associated with multiple advantages from pedagogical and formative points of view, including active involvement, as the player becomes the protagonist of learning and is stimulated to explore, experiment, and reason independently [29]. Another advantage is immediate feedback, as they provide a timely and accurate response on the player’s actions, allowing learning based on error and its correction. Other advantages include experiential learning related to scientific literacy [58], as they allow for real-world situations to be experienced and simulated, providing a direct and engaging educational experience; collaborative learning, as they can be used to foster cooperative learning, stimulating participation and information sharing even in educational settings between teachers and students [59,60]; and learning personalization [61], as they can be designed to adapt to each player’s cognitive needs and abilities, sparking and maintaining player interest and motivation and enhancing positive learning effects [32]. In addition, there is a positive relationship between the scenarios designed for serious games and the expected outcomes of the learning experience [62] considering, as part of this process, student engagement and academic performance [33].

Despite the potential of these tools, they still pose a challenge to the educational world [34]. It is important to place more emphasis on less formal and traditional models that are more suitable for teaching, which can be projected to the generation of students experiencing school today. Serious games, according to this perspective, represent an important resource for the world of training and education, and are facilitators for learning specific disciplines.

#### Soft Skills in the Labor World

Soft skills refer to a range of personal attributes, social abilities, and communication competencies that are necessary for success in the workplace. These skills, which are distinct from technical skills, include qualities such as positive attitudes, good communication, and strong interpersonal skills. They are also described as the “human skills” or “people skills” needed to effectively use technical knowledge and expertise in a professional setting [63].

Several studies have focused on identifying the key soft skills that are valuable in the labor market [64]. Several international studies have shown that soft skills are critical to our future success, and their importance is recognized by education providers, academics, human resource departments, and businesses [65-68].

This research aims to understand how companies and economic organizations that recruit, hire, and develop professionals at a medium to high-level view and value soft skills in their human resource development process [69].

According to the study, the most important soft skills for different groups, determined based on criteria such as significance, relevance, availability in the job market, and negative impact if lacking, are as follows: problem solving (for the group focused on achieving results), team working (for the group focused on mastering social skills), and time management (for the group focused on making progress in the workforce).

In order to examine the testability and relevance of certain soft skills, it is also useful to analyze communication and decision-making in greater depth. Additionally, problem solving may be linked to self-directed learning and communication for goal orientation, and both of these skills may be considered when discussing digital skills [70]. Therefore, it is advisable to carefully analyze these 5 skills, which were identified through a classic assessment. To assess communication [71], the communicative style (aggressive, passive, or assertive) [72-74] was adopted, and for problem solving, models, such as DMAIC (Define, Measure, Analyze, Improve, and Control) [75] and Problem Solving Inventory (PSI) [76], were used. Team working and related skills developed in group environments are highly sought after by recruiters when hiring [77]. The research was inspired by the Comprehensive Assessment of Team Member Effectiveness (CATME) [78], and for decision-making, the cognitive style (a person’s preferred way of thinking and tendency to adopt certain strategies more frequently) [79] was evaluated using the General Decision-Making Styles (GDMS) scale [80]. Time management, as theorized by Lakein [81] and others, was assessed using the time management behavior scale [82]. Soft skills are increasingly recognized as critical to future success and are valued by those in education, academia, and human resource departments. They also help organizations gain new customers, increase revenue, and reduce employee turnover, which is why employers are placing an increasing emphasis on these skills [66].

### Purpose of the Study and Origins

This study was conceived within the scope of the SWAG (Skills in Work-life Analysis Game) project. SWAG sets out to democratize the human resource world, through the use of intergenerational language, and it is inclusive and comprehensive of the needs, complexity, and peculiarities of today’s and tomorrow’s talents, connecting the right person to the right position. The SWAG team (involving Altomari Luca, Altomari Natalia, and Vigliaturo Daniele) was created in the context of the first cycle of “UniCaLab” (Contamination Lab of the University of Calabria) in 2019 (path concluded among the 3 winners) and achieved victory in Invitalia’s “#IoRestoAlSudHackathonTour*” contest in the same year.

In this work, a new assessment framework is presented that takes into account the needs of the labor market and is based on gamification, with a focus on the concept of “recrutainment” (a term combining “recruitment” and “entertainment”), which integrates cognitive and aptitude assessment methods into the personnel selection process [83] and is not only partially conceived as a subset of gamification, but also goes beyond it [84]. In this study, a serious game that assesses the most in-demand skills in the job market and provides feedback is illustrated, proposing a set of guidelines for the development of serious games while describing the game design process. The first trial of the game was conducted and positive results were achieved, paving the way for further studies.

The serious game Among the Office Criticality (AOC) was developed by the SWAG team as a desktop version with the technical support of Artémat.

## Methods

### Research Methodology and Proposal of a Serious Game

**Figure 1:**
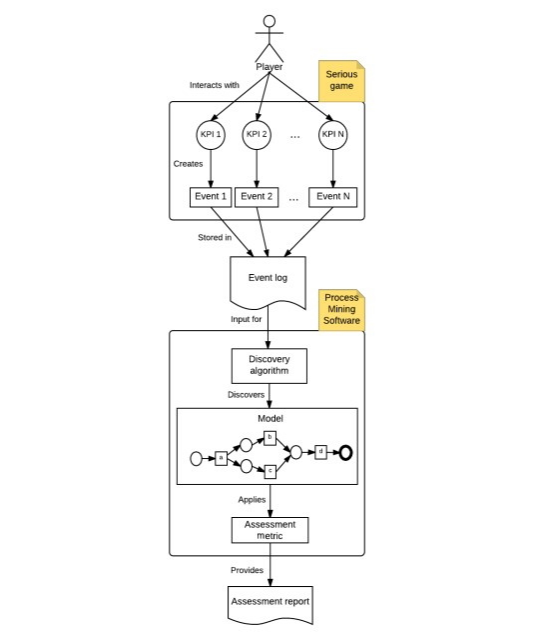
Architecture for skill assessment in serious games based on event sequence analysis. KPI: key performance indicator.

The purpose of this study was to develop a model that can identify and evaluate certain work-life skills (in this case, 5 soft skills that are in high demand in the job market) and provide feedback to the user who uses the tool. Gamification is particularly suitable for this purpose because it allows for the creation of tools that can provide quick, objective, and standardized assessments [85] after an initial design and implementation effort, while also allowing for a more informal approach to the person being evaluated [12,13]. The study presents the AOC serious game, which is able to detect the 5 soft skills mentioned above. The AOC serious game uses a set of sequence analysis techniques, which is known as process mining, to overcome the limitations of traditional skills assessment methods [85]. The user interacts with the game, which tracks key performance indicators (KPIs) based on the user’s choices at each decision point and saves them in an event log at the end of the game. These events and values are processed by process mining software, which generates an output (Figure 1) [85].

The software that analyzes the event log uses an algorithm to read the data in the log and create a model. This model is then used to apply the metrics of the specific evaluation tool to produce the final report.

AOC is a serious game that is structured as a tree graph, with decisional moments represented as nodes. Analyzing the user’s choices within the game involves identifying the pattern arcs that connect the nodes. However, analyzing all user actions within the game can cause scalability issues. To address this, the event log only records data related to decisional moments and the duration of the game, rather than all interactions. In this case, sequence analysis can be helpful in solving or mitigating the problem [85]. In the AOC game, metrics are based on a data set of fixed-length numerical vectors and an overall rating formula. These vectors are added together based on the entries in the event log to create a final vector, the values of which are then used in the formula to produce a numerical result. This result, as well as the individual values, can be easily translated into a final report that is provided to the user in both graphical and narrative forms. The design of serious game processes can be performed in 2 steps: context analysis and iterative concept use. The first step involves using user-centered design methods, which focus on the user’s intent, situation, activity, and characteristics. The second step involves testing the tool, once it has been defined and materialized as a prototype or mock-up, in order to improve it with a focus on attractiveness, motivation, and support for activities [86]. It is important to keep in mind some fundamental principles of gamification, such as freedom of choice, benefits and relevance, personalized experience, long-term interaction, unwanted side events, and ethical and legal elements [86]. The tree graph structure containing the decisions should also be considered.

### AOC Serious Game

AOC is a serious game that assesses 5 soft skills by immersing users in a simulated game context where they can use various tools as needed. The game context places the player directly into a company through a specific delivery at the beginning of the game. In the simulation, the player, who is starting their work experience, begins their journey at a fictious firm with an apprenticeship contract. At the start of the simulation, the user is presented with an organizational chart, and graphic and textual descriptions of the business context. Over the first 6 months of the game, the player must interact with other characters to complete tasks, deal with unexpected events, and make decisions, choosing the means of communication and working with superiors and other team members in order to achieve the best possible result at the end of the simulation. At the end of the game, based on the choices made, the user is presented with a scenario that reflects the path they took. The contract can be converted into permanent employment, extended for an additional 6 months, or terminated.

#### Serious Game Output

In the alpha version of the game, the user is presented with a series of decisions that are related to values for various soft skills. These values are individual variables that are combined into a resultant vector, denoted as R. The R values are then input into the overall evaluation formula, which produces a numerical result, designated as x. This result corresponds to an outcome that is related to the objective of the game. As the user makes decisions, the events of the game progress according to the graph. Each event occurs consecutively and presents the user with contextual choices that are as visual and immediate as possible (eg, a choice between 3 interactive texts). The values assigned to each decision are based on data from previous studies and the literature used to construct the game [87]. The game has a maximum duration of 30 minutes, after which the available time (displayed as a countdown) reaches zero. If the simulation is not completed at this point, the final result is the R vector, which does not consider any subsequent decisional moments and includes a penalty for time management. The “first-person” perspective is intentionally designed to increase the user’s engagement and encourage active interaction.

In 2017, van Laar et al [88] conducted a study that examined 75 studies on 21st century skills and found that problem solving and communication were the most commonly mentioned skills, with 24 and 22 quotes, respectively. In this study, communication was defined as the ability to transmit information effectively, while problem solving was defined as the ability to use ICT to understand and process problems and actively use knowledge to find solutions.

**Figure 2:**
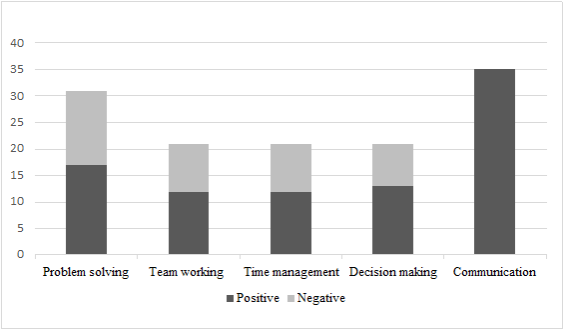
Positive and negative possible scores concerning every skill assessed by the serious game Among the Office Criticality. The ranges show the number of possible final resulting skill values.

The structure of the graph, the assignment of scores, and the analysis of these scores are all built using techniques from the Theory of Graphs, in line with theory and market research that emphasizes the importance of problem solving, which is tested in greater detail than the other skills. This is evident in the analysis of paths that maximize and minimize the variables associated with the skills, which has shown a range of 33 possible values for problem solving. The skills of team working, time management, and decision-making have 21 possible values and make up a total of 48% of the testing (Figure 2). The remaining 52% is composed of problem solving and communication, with this last skill requiring more attention as it is divided into 3 categories: passive communicative style, aggressive communicative style, and assertive communicative style. Variables used to identify the communicative style are also included in this percentage.

The slight differences in amplitude within the ranges can be attributed to both psychobehavioral factors and mathematical advantages. The psychobehavioral reason is that each communication style has the potential to generate unexpected events within interpersonal relationships (ordered: aggressive, passive, and assertive). The mathematical advantage is that the 3 variables can never be equal in any of the possible combinations. The outcome for the user consists of a final report that includes both graphical and narrative components (Table 1).The graphical component consists of a 2D diagram that incorporates a polygon, with each vertex representing the user's scaled final scores in the four soft skills: team working, time management, decision-making, and problem solving. This polygon describes a surface of amplitude that is directly proportional to the x value resulting from the overall evaluation formula. The textual component includes a result relating to the final goal (3 possible scenarios) and different sections describing the user’s score in each individual skill (low, medium, and high). In the graphical component, the dominant communication style is also explicitly highlighted (aggressive, passive, or assertive).

**Table 1:** Range width comparison showing “how much” a single skill is assessed.

Skill	Assessment, %
Problem solving	25.19
Team working	16.03
Time management	16.03
Decision-making	16.03
Communication	26.72

### Ethics Approval

Ethics approval for the research project, “Innovation in Teaching Methods” was granted by the Ethics Committee of the University of Calabria (COMITATO ETICO DI ATENEO - CEA) on May 9, 2019. The project, which was conducted within the University of Calabria, aimed to assess soft skills using the tool developed by SWAG, alongside human tutors. Adhering to the approved ethical guidelines, the research team ensured participant confidentiality and privacy throughout the data analysis process.

## Results

### Elaboration and Descriptive Statistical Analysis

**Figure 3:**
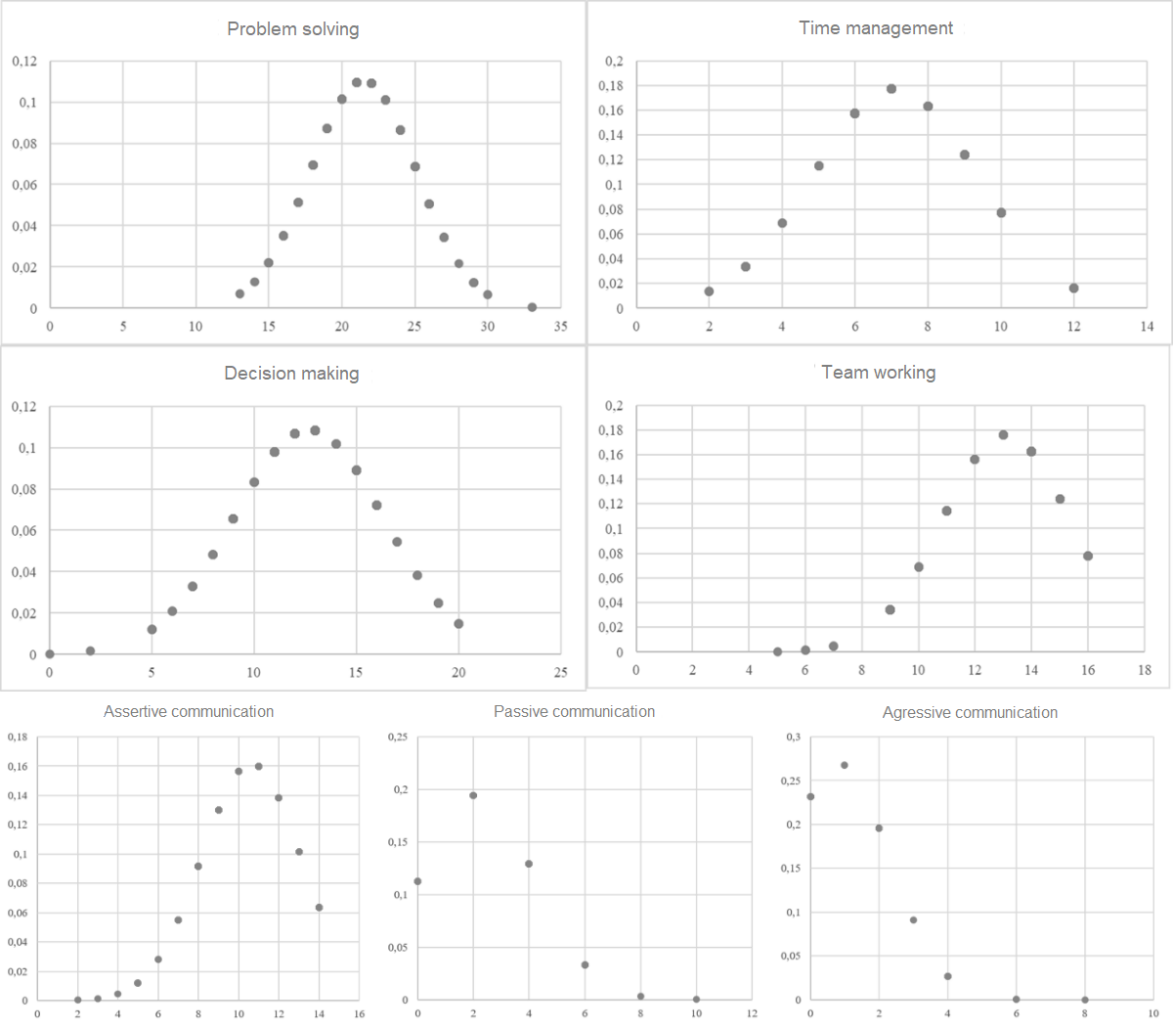
Normal distribution curves. The x-axis represents values, and the y-axis indicates probability in the normal curves.

To test the serious game, the researchers conducted a pilot study with 160 subjects from the University of Calabria (Italy) who were enrolled in the 2018/2019 academic year. Table 2 and Figure 3 show the results of the preliminary data screening and the normal curve of the collected data. According to the criteria of Curran, West, and Finch [89] (asymmetry greater than |1|=severe nonnormality; kurtosis greater than |3|=moderate nonnormality), the value for aggressive communication was close to being severely nonnormal in terms of asymmetry. This may be due to a small number of data points for individuals who had this type of communication as a result. While only 1 item showed these values, the results of the descriptive analyses can still be considered reliable.

**Table 2:** Normality indices related to the final scores obtained for each soft skill.

Skill	Score, mean (SD)	Asymmetry	Kurtosis
Problem solving	21.47 (3.615)	0.133	−0.109
Team working	13.10 (2.269)	−1.271	1.261
Time management	7.09 (2.251)	0.059	−0.165
Decision-making	12.68 (3.673)	−0.250	0.395
Assertive communication	10.63 (2.469)	−0.700	0.523
Passive communication	2.15 (2.050)	0.912	0.751
Aggressive communication	0.82 (1.479)	2.120	4.969

### First Try Out: Feedback Analysis

A trial of the AOC experiment was conducted, and at the end of the simulation, users were asked to complete a questionnaire about their experience. The data analysis showed a strong consistency between the user evaluation produced by AOC and the user’s self-awareness. Specifically, 88.3% (121/137) of responses to the question “Do you see yourself in the evaluation?” were at least “partly,” 70.1% (96/137) were at least “quite,” and 29.2% (40/137) were at least “yes.” It was also useful to analyze the users’ responses to the question “How would you describe the simulation in 3 adjectives?” The most common adjective used was “interesting,” with a frequency of 16.1% (63/392) of all adjectives, followed by “fun” (36/392, 9.2%), “useful” (23/392, 5.9%), “engaging” (22/392, 5.6%), “realistic” (22/392, 5.6%), “stimulating” (17/392, 4.3%), and “innovative” (16/392, 4.1%), only considering adjectives with more than 15 repetitions (Table 3).

**Table 3:** Top unique repetitions of adjectives given as feedback from the assessed users to the serious game “Among the Office Criticality” after an assessment session.

Adjective	Unique repetitions (N=392), n (%)
Interesting	63 (16.1)
Fun	36 (9.2)
Useful	23 (5.9)
Engaging	22 (5.6)
Realistic	22 (5.6)
Stimulating	17 (4.3)
Innovative	16 (4.1)
Sympathetic	15 (3.8)
Effective	10 (2.6)
Appealing	7 (1.8)
Constructive	7 (1.8)
Efficient	7 (1.8)
Rapid	6 (1.5)

We found it useful to group the adjectives used by users into clusters based on their focus during the simulation. These clusters were arbitrarily defined as “involvement” (cluster 1; 120/392, 30.6%), “pleasure” (cluster 2; 73/392, 18.6%), “functionality” (cluster 3; 71/392, 18.1%), “innovation” (cluster 4; 25/392, 6.4%), “truthfulness” (cluster 5; 23/392, 5.9%), “dynamism” (cluster 6; 15/392, 3.8%), “personal growth” (cluster 7; 14/392, 3.6%), “challenge” (cluster 8; 5/392, 1.3%), and “other” (43/392, 11.7%). In this study, the first, second, and third clusters accounted for 67.3% (264/392) of user responses in the survey administered after they played the serious game. We found that these clusters corresponded to other questionnaires conducted after serious games in the literature. A key factor considered, as reflected in the final feedback from users, was fidelity.

Fidelity, referring to the consistency of the game with the real world, is a crucial attribute for reality-based games as it enhances player immersion. Immersion refers to the player’s involvement in the “player’s world,” which is a crucial factor in motivating players to pursue “serious” goals [90]. However, it is important to balance immersion with entertainment elements, as previous studies have shown [27,91,92]. Fidelity can also contribute to the entertainment value of a game.

The enjoyment of playing a video game can increase motivation and lead players to find pleasure in the activities within the game, regardless of their achievements. This is especially relevant when the objectives of the game are perceived as enjoyable by the player [90].

Previous studies have found that user-perceived determinants after playing a serious game include feedback, ease of use and usability, and learning effectiveness, among others. This is consistent with the findings of this study, in which the analysis of the open-ended questionnaire taken by users at the end of the game revealed factors associated with the functionality cluster, such as ease of use and effectiveness [93]. Additionally, the enjoyment and presence-immersion factors identified in the study by Fokides et al [93] can be linked to the pleasure and involvement clusters.

According to a literature review (54 studies, 59 data sets, and 586 variables) conducted in 2019 by Baptista and Oliveira on gamification, the best predictors of acceptance for gamified technology were attitude, enjoyment, and usefulness on intention. The clusters of adjectives that were found to be most significant (involvement, pleasure, and functionality) in this study aligned with these findings, suggesting that the proposed serious game follows the trend of accepted gamification technology.

The results of the study showed that users were particularly interested in the level of engagement and interest generated by the simulation [94], as well as the entertainment value and overall emotional reactions (such as “interesting,” “engaging,” and “stimulating”). This aligns with the concept of recrutainment, which emphasizes the role of engagement in the recruitment process [95]. In addition, users were interested in the functionality of the assessment tool, as indicated by adjectives like “useful,” “effective,” and “efficient.” These results highlight the importance of designing the tool with users’ needs in mind. Overall, about half (193/392, 49.2%) of the adjectives in the involvement and pleasure clusters [86] supported the theory of user-centered design.

## Discussion

### Role of Gamification in Assessing Soft Skills for Talent Acquisition

This study proposes an immersive serious game model, called AOC, which aims to evaluate 5 important soft skills sought after in the job market, namely, problem solving, team working, time management, decision-making, and communication. The design of AOC was based on the literature and followed a user-centered design approach to create a user-friendly and intuitive environment. The output provided to the user holds significance as AOC was designed to generate value for both the evaluator and the evaluated subject. Therefore, it is essential to show areas of potential growth and increase the user’s self-awareness after the simulation. This study found that the proposed serious game model may be considered in line with existing literature. Nevertheless, it is worth acknowledging that this study is not without limitations. One such limitation is the relatively small sample size, which may have implications for the generalizability of the findings. To build upon and strengthen our findings, future studies with larger and more diverse samples are recommended.

Gamification is a viable solution to improve some aspects of personnel selection [95], particularly the concept of recrutainment, which focuses on the candidate’s involvement in the selection process [4,83]. Serious games are useful for both education and assessment, and are increasingly used in business and work contexts, as they help reduce selection time, avoid subjective evaluation [70], and reduce cognitive bias. They also allow for employer branding, which can positively influence the user’s opinion of the evaluator [84]. According to Diercks and Kupka [84], the use of self-assessment elements in selection can increase the success rate of achieving objectives to 93%, especially when employer branding and personnel marketing are properly carried out. The term “recrutainment,” which combines the words “recruiting” and “entertainment,” has gained popularity among Generation Y and refers to gamified, user-oriented, and simulation games that aim to improve the quality of suitable candidates through self-assessment procedures [84]. The use of gamification, combined with cognitive and aptitude assessment methods [57], is increasing in the field of recruitment and human resources in general. It is expected to triple from 2020 to 2025, with a focus on its use in personnel selection [95].

However, as noted by Kirovska et al in 2020 [95], implementing a gamification model in the recruitment process carries risks, including the need for constant monitoring. Therefore, many companies outsource it to third-party organizations to benefit from reduced hiring times and increased interaction levels, as well as to delegate risks. Attracting innovative talent is a goal for many organizations and allows them to consider characteristics and attitudes aligned with their brand’s aim. Gamification also encourages the user to do more than what is required [96], which can be a competitive advantage. In 2017, Korn et al [83] identified 42 applications (evaluating 60% of them) proposed by well-known organizations that use recrutainment, with a focus on the correlation between the profile and the hiring subject’s needs (job relatedness). They also emphasized that a low score in a single game does not necessarily mean poor user skills and that, in fact, each result could identify areas for improvement.

The human resource industry has come to fully understand and use gamification in the talent acquisition process, including from a strategic perspective. While the evaluation of technical skills is important and can be inferred from a candidate’s resume, transversal skills, which are intrinsic, innate, or developed through training and life experiences, are more difficult to objectively evaluate. This requires a significant amount of time and energy from human resource professionals [97]. In this context, gamification becomes important as it allows certain characteristics to emerge indirectly, which can be very complicated to discover otherwise [63]. Soft skills can make a significant difference in the business environment, and serious games are particularly effective at evaluating them because they can be assessed in various contexts and methods [12,13], including through self-assessment.

### Conclusions and Future Developments

This research presents AOC, a serious game designed to meet the needs of the job market and place the user at the center. Evaluation techniques consistent with the literature were used to create an interdisciplinary approach for adequately evaluating the user and adding value. Initial analysis suggested that the scores obtained by subjects during the serious game can be generalized to the general population. Additionally, feedback from a postgame questionnaire indicated that the game is engaging, enjoyable, functional, innovative, and realistic, and promotes personal growth. The feedback specifically emphasizes the engagement and interest sparked by the simulation, aligning with the principles of user-centered design and recrutainment theory.

In conclusion, we propose the methodologies used in the development of the serious game AOC as a framework for designing serious games that consider the key aspects of the user journey and their relevance to theory and traditional assessment.

Future work may include analyses of the data generated by the serious game, including skills of the studied sample, skill development over time, cross-analysis with other samples, and comparison with data from other serious games evaluating different work-life skills.

## Abbreviations

AI: artificial intelligence
AOC: Among the Office Criticality
ICT: information and communications technology

## References

[1] Huotari K, Hamari J (2012). Defining gamification: a service marketing perspective. MindTrek '12: Proceeding of the 16th International Academic MindTrek Conference.

[2] Hamari J, Ritzer G, Rojek C (2019). Gamification. The Blackwell Encyclopedia of Sociology.

[3] Deterding S, Dixon D, Khaled R, Nacke L (2011). From game design elements to gamefulness: defining "gamification". MindTrek '11: Proceedings of the 15th International Academic MindTrek Conference: Envisioning Future Media Environments.

[4] Csikszentmihalyi M (1990). Literacy and intrinsic motivation. Daedalus.

[5] Weisberg DS, Hirsh-Pasek K, Golinkoff RM (2013). Embracing complexity: rethinking the relation between play and learning: comment on Lillard et al. (2013). Psychol Bull.

[6] Hirsh-Pasek K, Golinkoff RM, Berk LE, Singer DG (2009). A Mandate for Playful Learning in Preschool: Presenting the Evidence.

[7] Oblinger DG (2004). The Next Generation of Educational Engagement. JIME.

[8] Callan RC, Bauer KN, Landers RN, Reiners T, Wood L (2015). How to Avoid the Dark Side of Gamification: Ten Business Scenarios and Their Unintended Consequences. Gamification in Education and Business.

[9] Morschheuser B, Henzi C, Alt R (2015). Increasing Intranet Usage through Gamification -- Insights from an Experiment in the Banking Industry.

[10] Morschheuser B, Riar M, Hamari J, Maedche A (2017). How games induce cooperation? A study on the relationship between game features and we-intentions in an augmented reality game. Computers in Human Behavior.

[11] Rajanen M, Rajanen D (2017). Usability Benefits in Gamification. CEUR Workshop Proceedings.

[12] Armstrong MB, Landers RN, Collmus AB, Gangadharbatla H, Davis D (2016). Gamifying Recruitment, Selection, Training, and Performance Management: Game-Thinking in Human Resource Management. Emerging Research and Trends in Gamification.

[13] Fetzer M, McNamara J, Geimer JL, Goldstein HW, Pulakos ED, Passmore J, Semedo C (2017). Gamification, Serious Games and Personnel Selection. The Wiley Blackwell Handbook of the Psychology of Recruitment, Selection and Employee Retention.

[14] Shute V, Ventura M, Bauer M, Zapata-Rivera D, Ritterfeld U, Cody M, Vorderer P (2009). Melding the power of serious games and embedded assessment to monitor and foster learning: flow and grow. Serious Games: Mechanisms and Effects.

[15] Shute V, Ventura M (2013). Stealth Assessment: Measuring and Supporting Learning in Video Games.

[16] Lamb RL, Annetta L, Firestone J, Etopio E (2018). A meta-analysis with examination of moderators of student cognition, affect, and learning outcomes while using serious educational games, serious games, and simulations. Computers in Human Behavior.

[17] Earp J, Ott M, Popescu M, Romero M, Usart M (2014). Supporting Human Capital development with Serious Games: An analysis of three experiences. Computers in Human Behavior.

[18] Azadegan A, Riedel JCKH, Baalsrud Hauge J, Ma M, Oliveira MF, Hauge JB, Duin H, Thoben KD (2012). Serious Games Adoption in Corporate Training. Serious Games Development and Applications. SGDA 2012. Lecture Notes in Computer Science, vol 7528.

[19] Ullah M, Amin SU, Munsif M, Safaev U, Khan H, Khan S, Ullah H (2022). Serious Games in Science Education. A Systematic Literature Review. Virtual Reality & Intelligent Hardware.

[20] Kim S, Song K, Lockee B, Burton J (2018). Students’ Perception of Gamification in Learning and Education. Gamification in Learning and Education. Advances in Game-Based Learning.

[21] Fleming TM, Bavin L, Stasiak K, Hermansson-Webb E, Merry SN, Cheek C, Lucassen M, Lau HM, Pollmuller B, Hetrick S (2016). Serious Games and Gamification for Mental Health: Current Status and Promising Directions. Front Psychiatry.

[22] Kazhamiakin R, Marconi A, Perillo M, Pistore M, Valetto G, Piras L, Avesani F, Perri N (2015). Using gamification to incentivize sustainable urban mobility.

[23] Church NJ, Iyer V (2018). The Future of Marketing: Staying competitive in a competitive world. Repositorio De La Red Internacional De Investigadores En Competitividad.

[24] Prestopnik NR, Tang J (2015). Points, stories, worlds, and diegesis: Comparing player experiences in two citizen science games. Computers in Human Behavior.

[25] Robson K, Plangger K, Kietzmann JH, McCarthy I, Pitt L (2015). Is it all a game? Understanding the principles of gamification. Business Horizons.

[26] Oostrom JK, Born MP, Serlie AW, van der Molen HT (2010). Webcam testing: Validation of an innovative open-ended multimedia test. European Journal of Work and Organizational Psychology.

[27] Giessen HW (2015). Serious Games Effects: An Overview. Procedia - Social and Behavioral Sciences.

[28] Georgiou K, Gouras A, Nikolaou I (2019). Gamification in employee selection: The development of a gamified assessment. Int J Select Assess.

[29] Bellotti F, Berta R, De Gloria A, Lavagnino E, Antonaci A, Dagnino F, Ott M, Romero M, Usart M, Mayer I (2014). Serious games and the development of an entrepreneurial mindset in higher education engineering students. Entertainment Computing.

[30] Altomari N, Valenti A (2023). Gamification as a tool for learning and assessment of soft skills at school. Form@re.

[31] Kara N (2021). A Systematic Review of the Use of Serious Games in Science Education. Contemporary Educational Technology.

[32] Moradi M, Noor NFBM (2022). The Impact of Problem-Based Serious Games on Learning Motivation. IEEE Access.

[33] Michael DR, Chen S (2006). Serious games : games that educate, train, and inform.

[34] Takeuchi LM, Vaala S (2014). Level up learning: A national survey on teaching with digital games. The Joan Ganz Cooney Center at Sesame Workshop.

[35] van Deursen A, van Dijk J (2009). Improving digital skills for the use of online public information and services. Government Information Quarterly.

[36] Dineen BR, Soltis SM, Zedeck S (2011). Recruitment: A review of research and emerging directions. APA handbook of industrial and organizational psychology, Vol. 2. Selecting and developing members for the organization.

[37] Ryan AM, Ployhart RE (2014). A century of selection. Annu Rev Psychol.

[38] Saini A, Garg V (2023). Transformation for Sustainable Business and Management Practices: Exploring the Spectrum of Industry 5.0.

[39] Liu-Lastres B, Wen H, Huang W (2022). A reflection on the Great Resignation in the hospitality and tourism industry. IJCHM.

[40] Rowley C (2023). Back to the future: post-pandemic work and management. PR.

[41] Chowdhury S, Dey P, Joel-Edgar S, Bhattacharya S, Rodriguez-Espindola O, Abadie A, Truong L (2023). Unlocking the value of artificial intelligence in human resource management through AI capability framework. Human Resource Management Review.

[42] Pérez J, Castro M, López G (2023). Serious Games and AI: Challenges and Opportunities for Computational Social Science. IEEE Access.

[43] Power Skills: Redefining Project Success. PMI.

[44] Cugelman B (2013). Gamification: what it is and why it matters to digital health behavior change developers. JMIR Serious Games.

[45] Pallavicini F, Pepe A, Clerici M, Mantovani F (2022). Virtual Reality Applications in Medicine During the COVID-19 Pandemic: Systematic Review. JMIR Serious Games.

[46] Kermavnar T, Visch VT, Desmet PMA (2023). Games in Times of a Pandemic: Structured Overview of COVID-19 Serious Games. JMIR Serious Games.

[47] Abd-Alrazaq A, Abuelezz I, Hassan A, AlSammarraie A, Alhuwail D, Irshaidat S, Abu Serhan H, Ahmed A, Alabed Alrazak S, Househ M (2022). Artificial Intelligence-Driven Serious Games in Health Care: Scoping Review. JMIR Serious Games.

[48] Schönbohm A, Zhang TV (2021). Evaluating the effectiveness of serious games in facilitating strategic decisions-making under COVID-19 crisis conditions. JWAM.

[49] Duch-Brown N, Gomez-Herrera E, Mueller-Langer F, Tolan S (2022). Market power and artificial intelligence work on online labour markets. Research Policy.

[50] Deranty J, Corbin T (2022). Artificial intelligence and work: a critical review of recent research from the social sciences. AI & Soc.

[51] Hagel J, Wooll M (2019). What is work?. Deloitte Review, issue 24.

[52] Mitchell J, Guile D, Bouezzeddine M (2022). Fusion Skills and Industry 5.0: Conceptions and Challenges. Insights Into Global Engineering Education After the Birth of Industry 5.0.

[53] Smaldone F, Ippolito A, Lagger J, Pellicano M (2022). Employability skills: Profiling data scientists in the digital labour market. European Management Journal.

[54] Heredia J, Castillo-Vergara M, Geldes C, Carbajal Gamarra FM, Flores A, Heredia W (2022). How do digital capabilities affect firm performance? The mediating role of technological capabilities in the “new normal”. Journal of Innovation & Knowledge.

[55] Akrim A (2022). Covid-19 Dan Kampus Merdeka Di Era New Normal (Ditinjau Dari Perspektif Ilmu Pengetahuan). Aksaqila Jabfung.

[56] Masenya TM, Aquino Jr PG, Jalagat Jr RC (2022). Integrating Talent and Knowledge Management Practices in the New Normal Business Environment: Developing Future Leaders in Public Sector Organizations. Navigating the New Normal of Business With Enhanced Human Resource Management Strategies.

[57] Budhwar P, Malik A, De Silva MTT, Thevisuthan P (2022). Artificial intelligence – challenges and opportunities for international HRM: a review and research agenda. The International Journal of Human Resource Management.

[58] Jung H, Lee C, Jhun Y (2014). Development and Application of an Evaluation Tool for Serious Games. JKAIE.

[59] Cheng M, Lin Y, She H, Kuo P (2016). Is immersion of any value? Whether, and to what extent, game immersion experience during serious gaming affects science learning. Br J Educ Technol.

[60] Connolly TM, Boyle EA, MacArthur E, Hainey T, Boyle JM (2012). A systematic literature review of empirical evidence on computer games and serious games. Computers & Education.

[61] Hess T, Gunter G (2013). Serious game-based and nongame-based online courses: Learning experiences and outcomes. Br J Educ Technol.

[62] Silva FGM (2019). Practical Methodology for the Design of Educational Serious Games. Information.

[63] Weber MR, Finley DA, Crawford A, Rivera D (2009). An Exploratory Study Identifying Soft Skill Competencies in Entry-Level Managers. Tourism and Hospitality Research.

[64] De Pietro O, Altomari N (2019). Uno strumento per misurare le Soft Skills degli insegnanti: risultati di uno studio pilota (A Tool to Measure Teachers’ Soft Skills: Results of a Pilot Study). Advances in Social Science and Culture.

[65] Dall’Amico E, Verona S (2015). Cross-country survey on soft skills required by companies to medium/high skilled migrants. Valorize High Skilled Migrants.

[66] Soft skills for business success Building Australia's future workforce. Deloitte.

[67] Rodríguez Martínez A, Sierra Sánchez V, Falcón Linares C, Latorre Cosculluela C (2021). Key Soft Skills in the Orientation Process and Level of Employability. Sustainability.

[68] van Laar E, van Deursen AJAM, van Dijk JAGM, de Haan J (2020). Determinants of 21st-Century Skills and 21st-Century Digital Skills for Workers: A Systematic Literature Review. SAGE Open.

[69] van Laar E, van Deursen AJ, van Dijk JA, de Haan J (2019). Determinants of 21st-century digital skills: A large-scale survey among working professionals. Computers in Human Behavior.

[70] Lin N (2014). Assessing Classroom Participation and Performance through Gamification Systems in Foreign Language Classrooms.

[71] Roberts KH, O'Reilly CA (1974). Measuring organizational communication. Journal of Applied Psychology.

[72] Brekelmans M, Levy J, Rodriguez R, Wubbels T, Levy J (1993). A typology of teacher communication style. Do You Know What You Look Like? Interpersonal Relationships in Education.

[73] Johnson NJ, Klee T (2016). Passive-Aggressive Behavior and Leadership Styles in Organizations. Journal of Leadership & Organizational Studies.

[74] Pipaş MD, Jaradat M (2010). Assertive Communication Skills. JASO.

[75] de Koning H, de Mast J (2006). A rational reconstruction of Six‐Sigma's breakthrough cookbook. International Journal of Quality & Reliability Management.

[76] Heppner PP, Witty TE, Dixon WA (2016). Problem-Solving Appraisal and Human Adjustment. The Counseling Psychologist.

[77] American Management Association (2010). AMA (2010) Critical Skills Survey: Executive Summary.

[78] Loughry ML, Ohland MW, DeWayne Moore D (2016). Development of a Theory-Based Assessment of Team Member Effectiveness. Educational and Psychological Measurement.

[79] Antonietti A, Scurati C (2003). Development-learning contexts as school scenarios. Infanzia scenari di scuola.

[80] Scott SG, Bruce RA (2016). Decision-Making Style: The Development and Assessment of a New Measure. Educational and Psychological Measurement.

[81] Lakein A (1973). How to Get Control of Your Time and Your Life.

[82] Macan TH (1994). Time management: Test of a process model. Journal of Applied Psychology.

[83] Korn O, Brenner F, Börsig J, Lalli F, Mattmüller M, Müller A, Freund L, Cellary W (2018). Defining Recrutainment: A Model and a Survey on the Gamification of Recruiting and Human Resources. Advances in The Human Side of Service Engineering. AHFE 2017. Advances in Intelligent Systems and Computing, vol 601.

[84] Diercks J, Kupka K (2014). Recrutainment: playful approaches in personnel marketing and selection.

[85] Hernández JAC, Duarte MP, Dodero JM (2017). An architecture for skill assessment in serious games based on Event Sequence Analysis. TEEM 2017: Proceedings of the 5th International Conference on Technological Ecosystems for Enhancing Multiculturality.

[86] Marache-Francisco C, Brangier E (2013). Process of gamification. From the consideration of gamification to its practical implementation. Proceedings of the 6th International Conference on Advances in Human-oriented and Personalized Mechanisms, Technologies, and Services CENTRIC.

[87] de Mast J, Lokkerbol J (2012). An analysis of the Six Sigma DMAIC method from the perspective of problem solving. International Journal of Production Economics.

[88] van Laar E, van Deursen AJ, van Dijk JA, de Haan J (2017). The relation between 21st-century skills and digital skills: A systematic literature review. Computers in Human Behavior.

[89] Curran PJ, West SG, Finch JF (1996). The robustness of test statistics to nonnormality and specification error in confirmatory factor analysis. Psychological Methods.

[90] Stofella A, Fadel LM, Bernardes O, Amorim V (2022). Fidelity and Play Model: Balancing Seriousness. Handbook of Research on Promoting Economic and Social Development Through Serious Games.

[91] Laamarti F, Eid M, El Saddik A (2014). An Overview of Serious Games. International Journal of Computer Games Technology.

[92] De Gloria A, Bellotti F, Berta R (2014). Serious Games for education and training. IJSG.

[93] Fokides E, Atsikpasi P, Kaimara P, Deliyannis I (2019). Let players evaluate serious games. Design and validation of the Serious Games Evaluation Scale. ICG.

[94] Kupka K, Diercks J, Kupka K (2013). Online-Assessments im Recrutainment-Format: Wie gefällt das eigentlich den Bewerbern in der echten Auswahlsituation?. Recrutainment.

[95] Kirovska Z, Josimovski S, Kiselicki M (2020). Modern trends of recruitment - introducing the concept of gamification. Journal of Sustainable Development.

[96] Simpson P, Jenkins P Gamification and Human Resources: an overview. Brighton Business School.

[97] Schmitt N (2014). The Oxford Handbook of Personnel Assessment and Selection.

